# Oxymatrine protects articular chondrocytes from IL-1β-induced damage through autophagy activation via AKT/mTOR signaling pathway inhibition

**DOI:** 10.1186/s13018-024-04667-2

**Published:** 2024-03-11

**Authors:** Jinying Lu, Jiang Bian, Yutong Wang, Yan Zhao, Xinmin Zhao, Gao Wang, Jing Yang

**Affiliations:** 1 Department of Biochemistry and Molecular Biology, Basic Medical College, Jinzhou Medical University, No.40, Section 3 Songpo Road, Linghe District, Jinzhou, Liaoning, 121001 China; 2 Provincial Key Laboratory of Cardiovascular and Cerebrovascular Drug Basic Research, Jinzhou Medical University, No.40, Section 3 Songpo Road, Linghe District, Jinzhou, Liaoning, 121001 China

**Keywords:** Oxymatrine, interleukin-1β, Chondrocytes, AKT/mTOR pathway, Autophagy

## Abstract

**Background:**

Osteoarthritis (OA) is a common degenerative joint disease characterized by persistent articular cartilage degeneration and synovitis. Oxymatrine (OMT) is a quinzolazine alkaloid extracted from the traditional Chinese medicine, matrine, and possesses anti-inflammatory properties that may help regulate the pathogenesis of OA; however, its mechanism has not been elucidated. This study aimed to investigate the effects of OMT on interleukin-1β (IL-1β)-induced damage and the potential mechanisms of action.

**Methods:**

Chondrocytes were isolated from Sprague-Dawley rats. Toluidine blue and Collagen II immunofluorescence staining were used to determine the purity of the chondrocytes. Thereafter, the chondrocytes were subjected to IL-1β stimulation, both in the presence and absence of OMT, or the autophagy inhibitor 3-methyladenine (3-MA). Cell viability was assessed using the MTT assay and SYTOX Green staining. Additionally, flow cytometry was used to determine cell apoptosis rate and reactive oxygen species (ROS) levels. The protein levels of AKT, mTOR, LC3, P62, matrix metalloproteinase-13, and collagen II were quantitatively analyzed using western blotting. Immunofluorescence was used to assess LC3 expression.

**Results:**

OMT alleviated IL-1β-induced damage in chondrocytes, by increasing the survival rate, reducing the apoptosis rates of chondrocytes, and preventing the degradation of the cartilage matrix. In addition, OMT decreased the ROS levels and inhibited the AKT/mTOR signaling pathway while promoting autophagy in IL-1β treated chondrocytes. However, the effectiveness of OMT in improving chondrocyte viability under IL-1β treatment was limited when autophagy was inhibited by 3-MA.

**Conclusions:**

OMT decreases oxidative stress and inhibits the AKT/mTOR signaling pathway to enhance autophagy, thus inhibiting IL-1β-induced damage. Therefore, OMT may be a novel and effective therapeutic agent for the clinical treatment of OA.

## Background

Osteoarthritis (OA) is a common degenerative disease characterized by the breakdown of the cartilage matrix, chondrocyte hypertrophy, inflammation of the synovial membrane, and osteophyte formation in joints [1]. As of 2021, more than 22% of individuals above 40 years of age have knee OA [2]. OA has a significant impact on functional impairment and disability, with 80% of having limitations in movement and 25% having difficulty in performing their major daily activities [3]. Historically, OA treatment has focused on managing pain and inflammation using nonsteroidal anti-inflammatory drugs and other medications. However, these therapeutic approaches have proven to be inadequate for providing satisfactory patient outcomes. Consequently, there is an urgent need to explore alternative treatment options for individuals with OA [4].

Chondrocytes are the main cells in articular cartilage and play an important role in maintaining normal physiological functions and cartilage morphology. In OA, the degradation of the extracellular matrix (ECM) and the apoptosis of chondrocytes are two crucial pathogenic events [5, 6]. Autophagy is a self-degradation process and an important protective mechanism against cartilage degeneration and apoptosis. Autophagy serves as a protective mechanism for chondrocytes, preventing apoptosis and cartilage degeneration. Additionally, it enhances the functionality of the cartilage [7–14]. Studies have linked the pathological processes of OA to reduced autophagy in chondrocytes [11]. Impaired autophagy is associated with the development and increased severity of OA [15], and improving autophagy can have therapeutic benefits in OA [16]. Inflammatory cytokines, such as interleukin-1β (IL-1β) can suppress autophagy and contribute to cartilage degeneration in OA [17].

Oxymatrine (OMT) is a quinzolazine alkaloid extracted from the traditional Chinese medicine matrine. It exhibits lower toxicity than matrine, making it an attractive option for further studies. It is currently used as an adjuvant drug for hepatitis, tumors, and other clinical diseases [18, 19]. In recent years, there has been significant interest in the use of OMT because of its effects on oxidative stress, inflammation, and apoptosis [20–22]. Studies have shown that OMT plays a protective role in OA by regulating chondrocyte homogeneity and inhibiting osteoclast generation [23, 24]. It also plays a protective role in chondrocytes by inhibiting NF-κB signaling [24]. These findings suggest that OMT has a potential therapeutic effect on OA. The AKT/mTOR pathway is a well-known autophagy-related signaling pathway that attenuates autophagy when activated [25]. OMT treatment of SW982 human synovial sarcoma cells resulted in reduced expression levels of phosphorylated AKT and phosphorylated mTOR. Based on these findings, we hypothesize that OMT induces autophagy through the same mechanism in OA. However, the mechanisms by which OMT may help regulate the pathogenesis of OA have not been elucidated.

This study aimed to investigate the protective effects of OMT on IL-1β induced chondrocytes and examine its impact on the AKT/mTOR signaling pathway and autophagy. Towards this goal, rat chondrocytes cultured in vitro were used to create a model of IL-1β-induced chondrocyte damage to mimic OA at the cellular level.

## Methods

### Chondrocyte isolation and cell culture

Articular cartilage was obtained from the knees of 8-week-old specific pathogen-free Sprague-Dawley rats. The cartilage was separated from the subchondral bone and cut into small pieces using sterile scissors and then stored in sterile phosphate-buffered saline (PBS) containing 1% penicillin–streptomycin solution. Primary chondrocytes were isolated by digestion with 0.25% Trypsin-EDTA Solution (Beyotime, Shanghai, China) for 0.5 h at 37 °C in a thermostatic shaker and Type IV collagenase (1 mg/mL, GenView) for 3 h at 37 °C. The cells were resuspended in DMEM/F12 containing 10% fetal bovine serum (GenView). Chondrocytes were cultured in incubator at 37 °C in 5% CO_2_. The cells were passed to 2–3 generations for subsequent experiments.

All animal experimental procedures and protocols were conducted in conformity with the principles of the Institutional Animal Care and Use Committee of Jinzhou Medical University, approval number 2022031001. All efforts were made to minimize the number of animals used and their suffering.

### Toluidine blue staining

The second-generation chondrocytes were seeded into a 24-hole plate and cultured for 72 h. The cells were then washed with PBS, fixed in 4% neutral formalin for 30 min, and stained with 1% toluidine paraformaldehyde for 1 h at 25 °C followed by 2 washes with PBS. Subsequently, the cells were stained with 1% toluidine blue for 2 h at 25 ± 3 °C [26]. Images were captured using an inverted fluorescence microscope after the removal of the staining dye.

### Immunohistochemical staining

The second-generation chondrocytes were fixed with 4% paraformaldehyde in 24-well plates for 30 min, washed three times with PBS, permeabilized for 20 min with 0.5% Triton X-100 in PBS at 4 °C, blocked for 30 min with 3% BSA, and probed overnight at 4 °C with anti-Collagen II (1:100) or LC3(1:100) in 3% BSA. After three rinses with 3% BSA, the cells were probed for 3 h with a secondary antibody (EarthOx, 1:50) in 3% BSA at room temperature, and the nuclei were stained with DAPI for 10 min [27]. Images were captured using an inverted fluorescence microscope (Nikon).

### Experimental grouping and drug administration

To assess the effectiveness of OMT (Biopurify, Chengdu, China), the second-generation chondrocytes were divided into the control group (Con group), the IL-1β (ABclonal) group, and the IL-1β + OMT (0.25, 0.50, 1.00 mg/mL OMT) group. In the control group, the chondrocytes were cultured in complete medium for 24 h. In the IL-1β group, the chondrocytes were cultured in complete medium containing 10 ng/mL IL-1β for 24 h. In the IL-1β + OMT group, the chondrocytes were initially incubated in complete culture medium containing 0.25, 0.50, or 1.00 mg/mL OMT for 2 h. Subsequently, IL-1β was added to achieve a final concentration of 10 ng/mL in the culture medium, and the chondrocytes were further incubated for 24 h.

To investigate the role of autophagy in the effectiveness of OMT, chondrocytes were treated with 3-MA(5 mM) (MCE, Shanghai, China) for 2 h [28], followed by the addition of complete culture medium containing 1 mg/mL OMT for an additional 2 h. Subsequently, IL-1β was introduced into the complete culture medium to attain a final concentration of 10 ng/mL, and the cells were further incubated for a total of 24 h.

### Cell viability assay

Cell viability was determined using the MTT assay. Chondrocytes were seeded in 96-well plates (5000 cells/well) and treated as experimental groups for drug administration. Chondrocytes were incubated with the MTT reagent (Solarbio, Beijing, China) at 37 °C for 4 h. Afterward, dimethyl sulfoxide (Solarbio) was added to dissolve the formazan product, and absorbance at 570 nm was examined using a microplate reader (Allsheng, Hangzhou, China). Three independent assays were performed.

### SYTOX green staining

SYTOX Green (Baiao Laibo, Beijing, China) is an excellent green fluorescent nuclear and chromosome counterstain that is impermeant to live cells but penetrates the compromised membrane characteristics of dead cells, making it a useful indicator of dead cells within a population. Chondrocytes were cultured in 12-well plates for 24 h and subjected to the experiments and drug administration. Then, cells were washed three times with PBS, and 1 µM SYTOX Green dead cell stain was added to each hole and mixed in the dark for 10 min at room temperature.

### Apoptosis assays

The apoptosis rate was evaluated using the Annexin V-FITC/PI (4 A Biotech, China) assay according to the manufacturer’s instructions. Chondrocytes were plated in 6-well plates and subjected to the experiments and drug administration. Following treatment, the cells were collected and washed with PBS and then resuspended in 1 mL binding buffer. Thereafter, 5 µL Annexin V-FITC was added to the cell suspension, and the cells were further incubated for 5 min at room temperature in the dark. Further, 10 µL PI and 400 µL PBS were added to the cell suspension. Cell fluorescence was assessed using flow cytometry within 1 h.

### Detection of reactive oxygen species

Reactive oxygen species (ROS) levels were evaluated using an ROS Assay Kit (Beyotime, Shanghai, China). Chondrocytes were plated in 6-well plates and subjected to the experiments and drug administration. The cultured chondrocytes were initially washed twice with 1×PBS, and then ROS levels were detected according to the manufacturer’s instructions. Cell fluorescence within 1 h was assessed using flow cytometry.

### Western blot

Cells were lysed in a lysis buffer containing phenylmethanesulfonyl fluoride (RIPA: PMSF = 100:1; Beyotime, Shanghai, China). The lysates were centrifuged at 12,000 ×*g* for 20 min at 4 °C, after which protein concentrations were measured using a Lowry method. Samples were separated using 10% sodium dodecyl sulfate-polyacrylamide gel electrophoresis, transferred to polyvinylidene fluoride membranes, and sealed for 2 h. Blots were probed overnight at 4 °C with appropriate antibodies and then incubated for 1 h with appropriate secondary antibodies (ABclone, 1:8000). The proteins were visualized using enhanced chemiluminescence (Tanon, Beijing, China). The primary antibodies used were specific for β-actin (AC004, ABclone, 1:50000), matrix metalloproteinase-13 (MMP-13)(18,165,Proteintech, 1:1000), Collagen II (ABS130072, Absin, 1:1000), AKT (ABS130889, Absin, 1:1000), mTOR (AP0115, ABclone, 1:1000), p62 (A11483, ABclone, 1:1000), and LC3B (A19665, ABclone, 1:1000).

### Statistical analysis

Data are expressed as the mean ± standard deviation. One-way analysis of variance followed by Tukey’s test was to compare among three or more groups. All experiments were performed at least three times. All statistical analyses were performed using GraphPad Prism 9.0 (GraphPad Software Inc, California, USA). *P* < 0.05 was considered statistically significant.

## Results

### Identification of chondrocytes

**Fig. 1:**
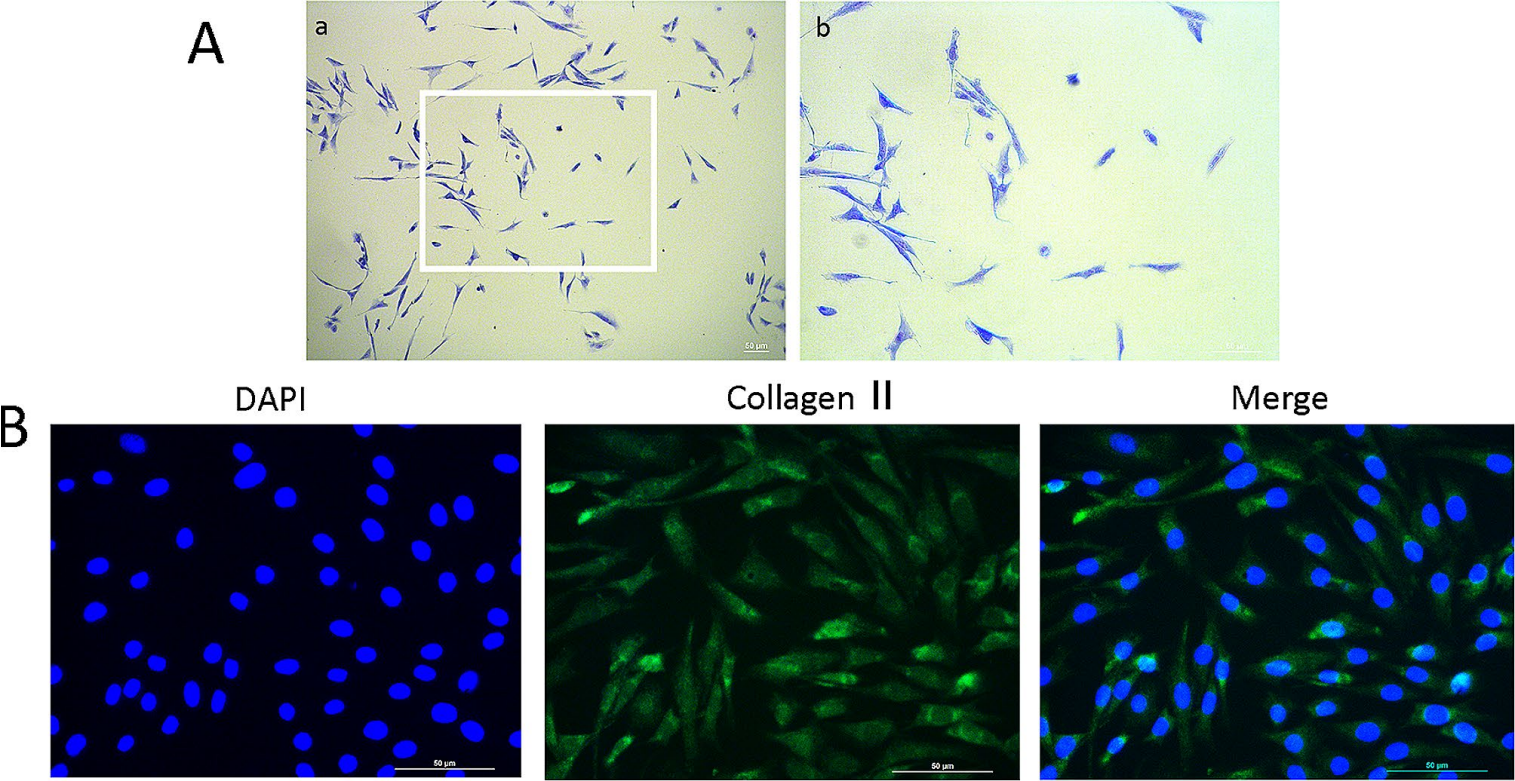
Morphological observations and identification of rat chondrocytes. (**a**) Toluidine blue-stained chondrocytes are observed under an inverted-phase contrast microscope. (**b**) Collagen II immunofluorescence-stained cultured chondrocytes are observed under fluorescence microscope. Scale bar = 50 μm

The main indicator of chondrocytes is proteoglycans, which can be stained with toluidine blue to appear blue-purple. In this study, the second-generation chondrocytes exhibited a blue-purple color, along with an elongated and spindle-shaped morphology, confirming their identity as chondrocytes (Fig. 1A). To further validate the isolated cells and assess their purity, we performed immunofluorescence staining for type II collagen, a chondrocyte-specific marker. Over 98% of cultured cells stained positive for collagen II, indicating their suitability for subsequent experiments (Fig. 1B).

### OMT improves IL‑1β treated chondrocyte viability

**Fig. 2:**
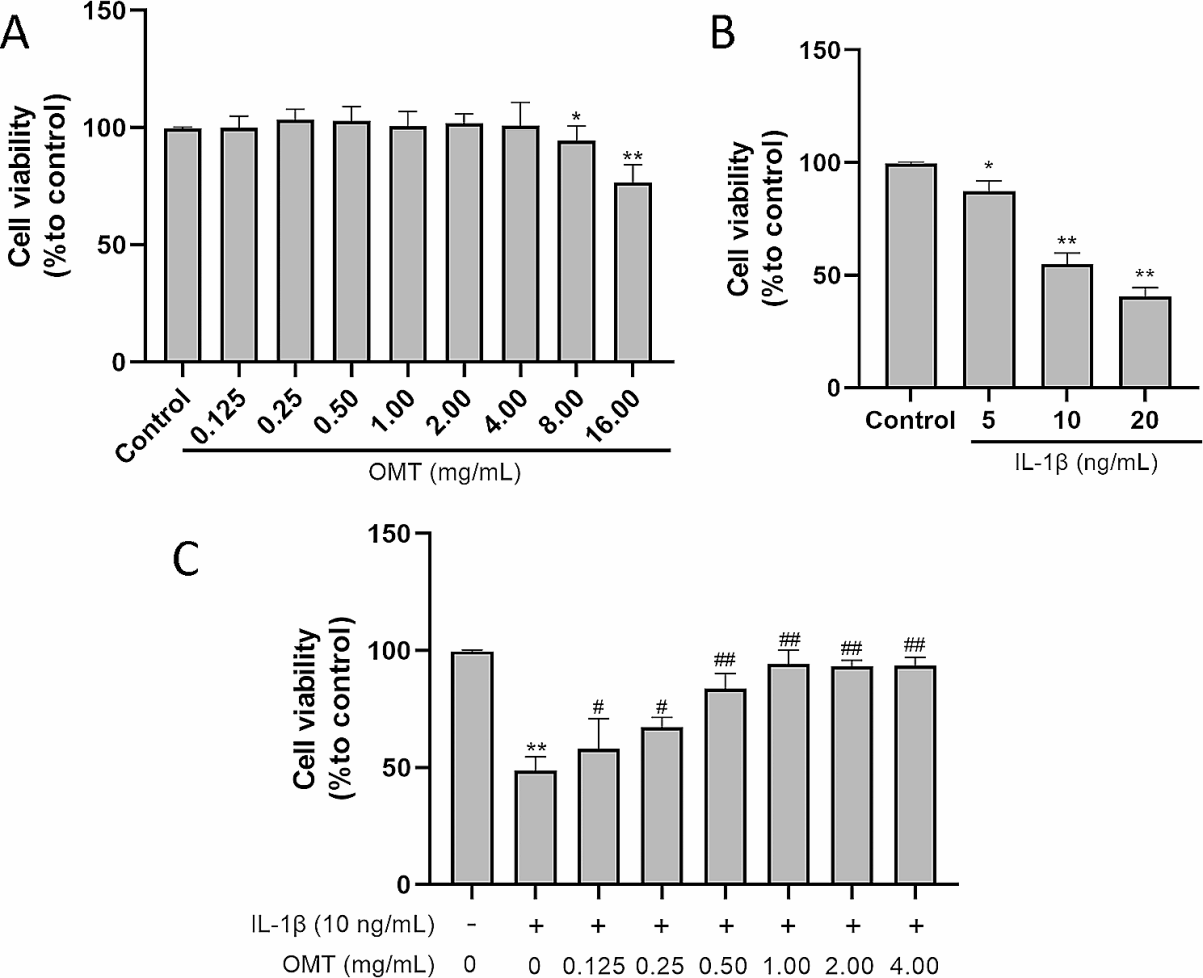
OMT improves IL-1β treated chondrocyte viability. (**a**) The cell viability of chondrocytes treated with different concentrations of OMT for 24 h as detected with the MTT assay. (**b**) The cell viability of chondrocytes treated with different concentrations of IL-1β for 24 h as detected with the MTT assay. (**c**) The effect of OMT on cell viability of chondrocytes treated with IL-1β for 24 h as detected with the MTT assay. Data are presented as the mean ± SD, *n* = 9 **P < 0.01, *P < 0.05 versus Con group; ^##^*P* < 0.01, ^#^*P* < 0.05 versus IL-1β group

To verify the toxicity of OMT, chondrocytes were cultured with different concentrations of OMT, and cell viability was measured using the MTT assay. The results are shown in Fig. 2A. OMT exerted no significant cytotoxic effects at concentrations below 4 mg/mL. To determine the optimal concentration of IL-1β injury for chondrocytes, cell viability was assessed following a 24-hour culture with 5, 10, and 20 ng/mL of IL-1β. Notably, the results revealed that the cell viability was close to 50% in the group treated with 10 ng/mL of IL-1β, suggesting it to be the most suitable concentration. Subsequent experiments were performed using this concentration, and the corresponding results are shown in Fig. 2B. To assess the protective effects of OMT, chondrocytes were pretreated with varying concentrations of OMT (ranging from 0.125 mg/mL to 4.00 mg/mL) for 2 h. Thereafter, the chondrocytes were subjected to injury induced by 10 ng/mL IL-1β for 24 h. The results demonstrated that compared with the IL-1β group, the OMT group showed significantly higher cell viability that increased in parallel with the increasing concentration of OMT (*P* < 0.01 or 0.05). However, no further increase in cell viability was observed beyond a concentration of 1.00 mg/mL. To establish a meaningful dose-response relationship, subsequent experiments were conducted using three doses: 0.25, 0.50, and 1.00 mg/mL (Fig. 2C).

### OMT mitigates IL-1β-induced damage in chondrocytes

**Fig. 3:**
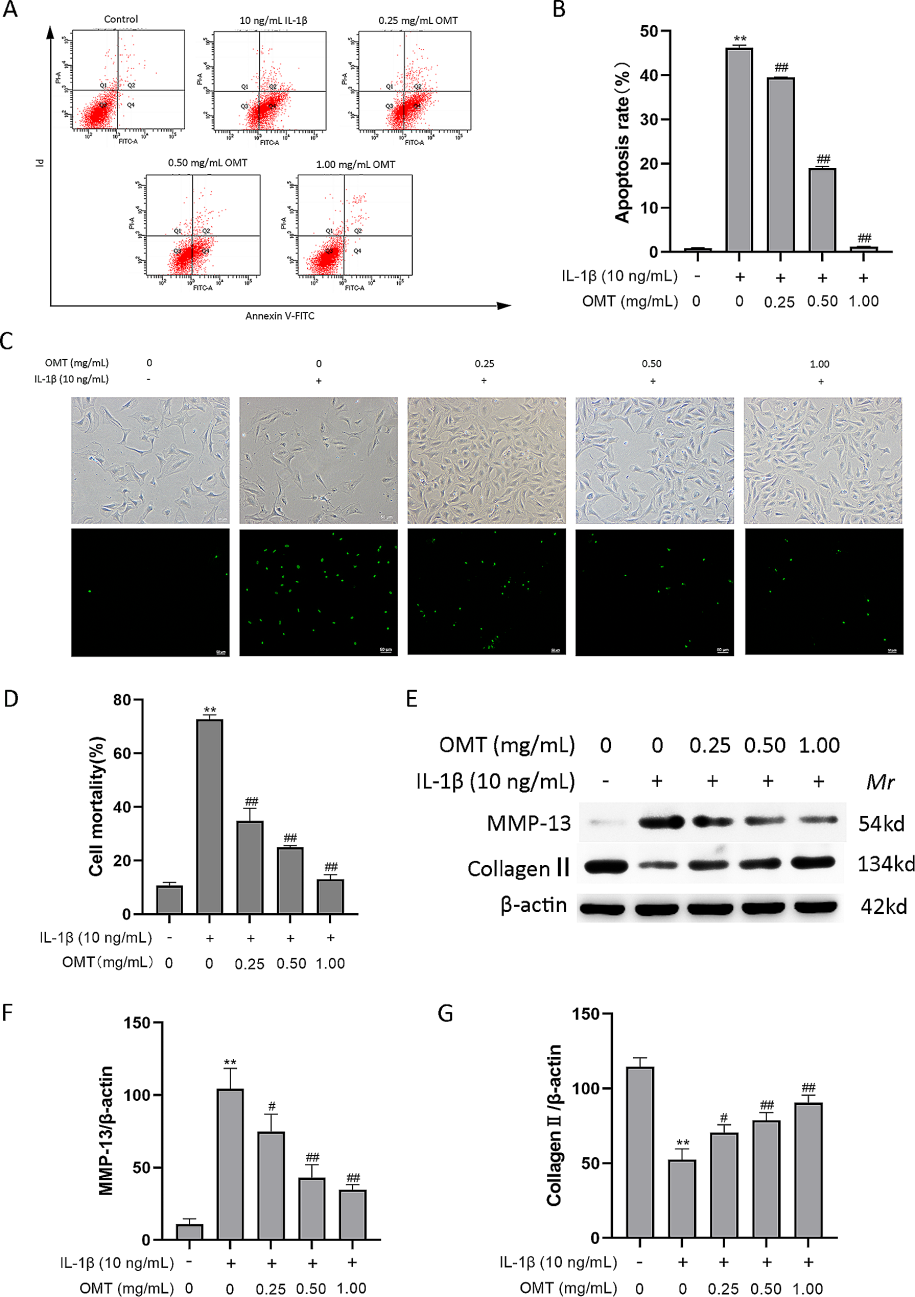
OMT mitigates IL-1β-induced injury in chondrocytes. (**a, b**) The cell apoptosis rates of chondrocytes as detected with Annexin V-FITC/PI assay using flow cytometry. (**c, d**) The cell damage rate of chondrocytes as detected with SYTOX Green staining. (**e**–**g**) The protein levels of collagen II and MMP-13 as detected with Western blot. Data are presented as the mean ± SD. A, B: *n* = 3/group; D–G: *n* = 3/group. **P < 0.01, *P < 0.05 versus Con group; ^##^*P* < 0.01, ^#^*P* < 0.05 versus IL-1β group

Cartilage damage caused by chondrocyte apoptosis plays a crucial role in OA [29]. To assess the extent of apoptosis, we conducted an Annexin V-FITC/PI assay using flow cytometry. The results indicate that the apoptosis rates of chondrocytes increased following IL-1β treatment (Fig. 3A and B). However, upon the addition of OMT at concentrations of 0.25, 0.50, and 1.00 mg/mL, the percentage of cells undergoing IL-1β-induced apoptosis decreased significantly from 46.23 ± 0.57% to 39.53 ± 0.06%, 19.03 ± 0.31%, and 1.23 ± 0.06%, respectively. Furthermore, SYTOX Green staining revealed increased chondrocyte damage in IL-1β-induced chondrocytes, and this was attenuated by OMT treatment (Fig. 3C, D). The degradation of the cartilage matrix caused by IL-1β is facilitated by catabolic enzymes such as MMPs, among which MMP-13 plays a significant role by breaking down the primary constituent of the extracellular matrix. Collagen II, a key component of the cartilage matrix, frequently undergoes degradation and reduction in cartilage affected by OA [30]. Western blot analysis in this study demonstrated that OMT increased the protein levels of collagen II and decreased those of MMP-13 (Fig. 3E-G).

### OMT decreases ROS levels in chondrocytes treated with IL‑1β

**Fig. 4:**
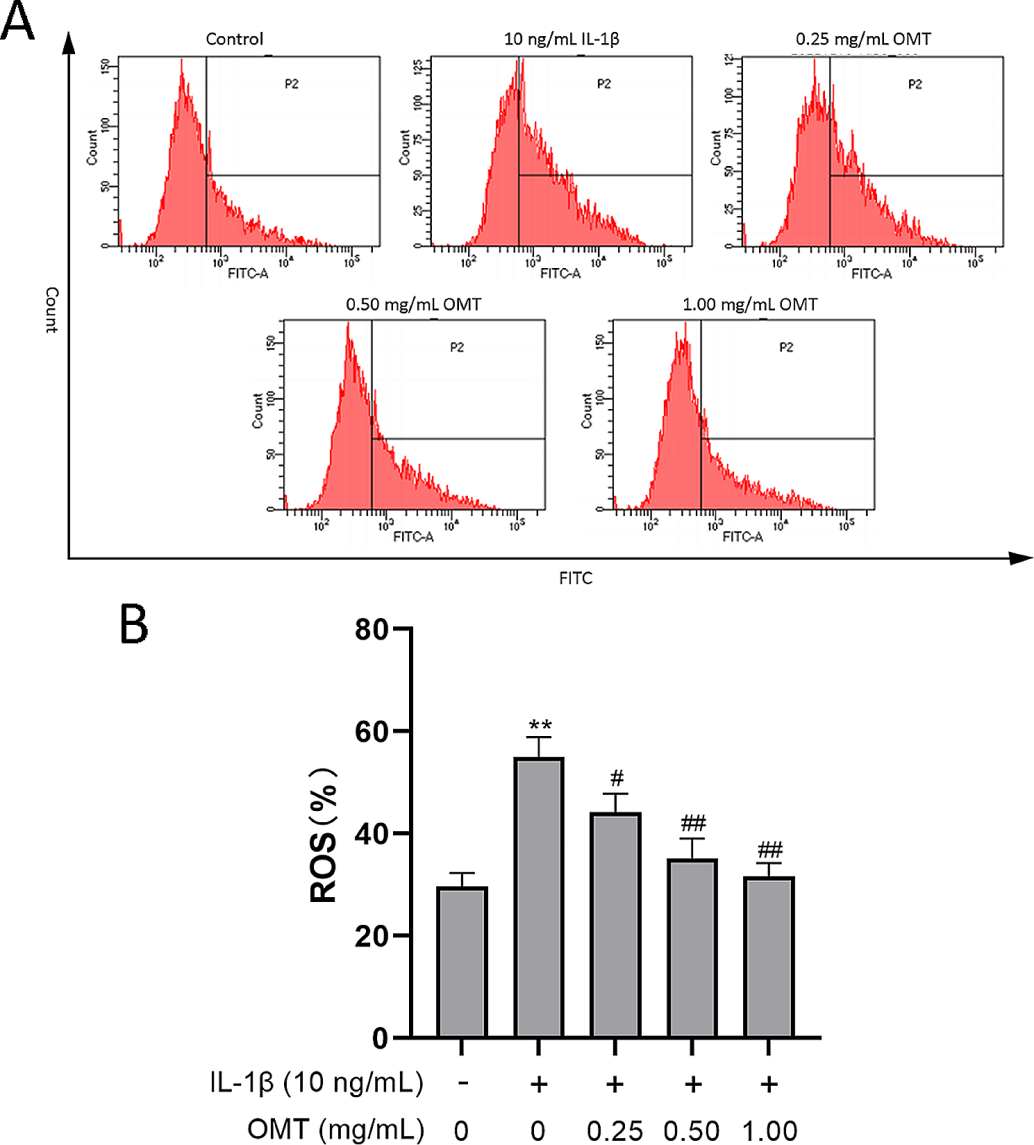
OMT decreases ROS levels in chondrocytes treated with IL‑1β. (**a, b**) The ROS level as assessed with the DCFH-DA method using flow cytometry. Data are presented as the mean ± SD, *n* = 3/group. **P < 0.01, *P < 0.05 versus Con group; ^##^*P* < 0.01, ^#^*P* < 0.05 versus IL-1β group

In this study, DCFH-DA was chosen to demonstrate whether OMT attenuated the levels of IL-1β-induced ROS in chondrocytes. The results showed that OMT at concentrations of 0.25, 0.50, and 1.00 mg/mL decreased ROS production (Fig. 4A) by 48.4%, 39.6%, and 34.6%, respectively (Fig. 4B). Further, OMT protected chondrocytes from an IL-1β-induced increase in ROS level.

### OMT decreases AKT/mTOR signaling pathway and increases autophagy in chondrocytes treated with IL‑1β

**Fig. 5:**
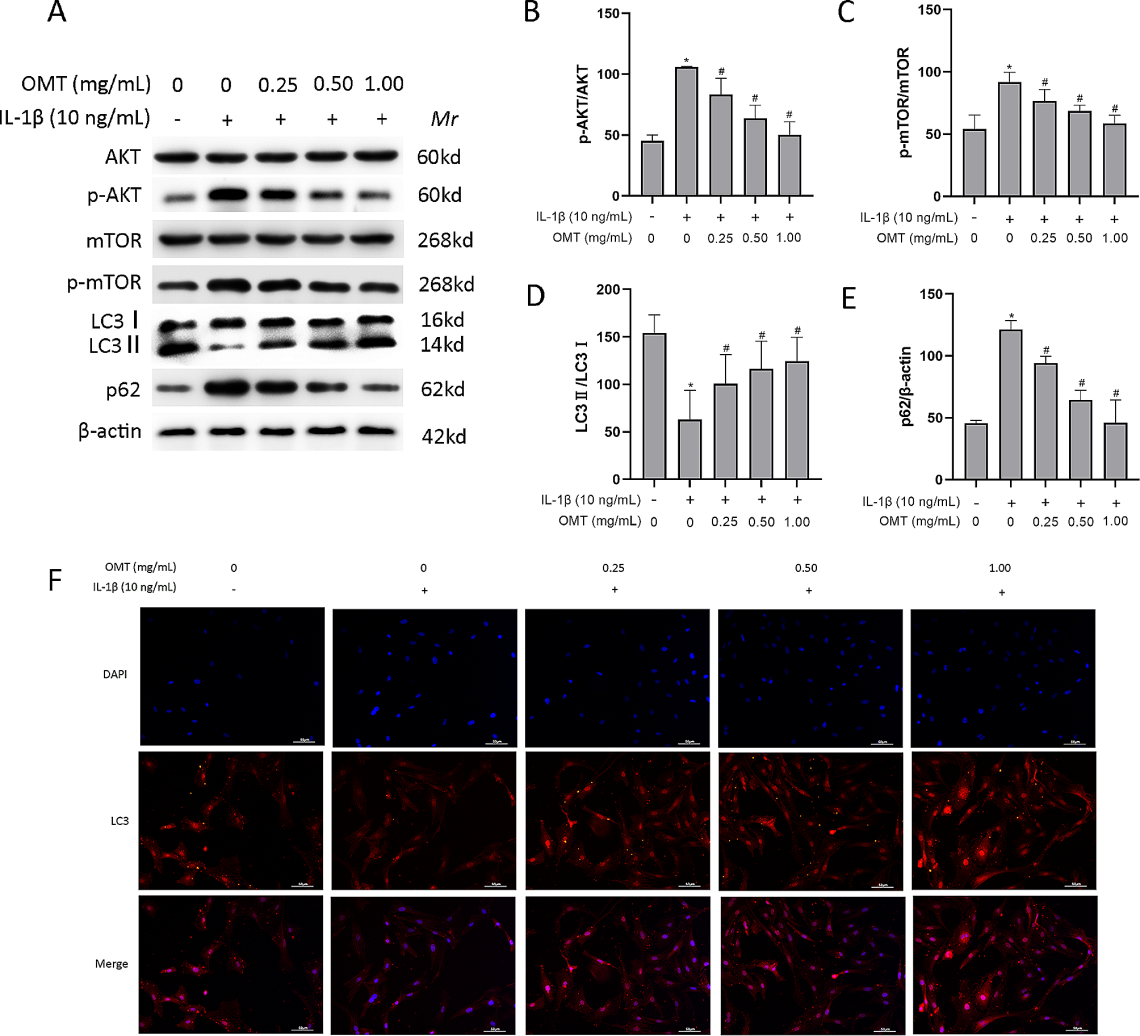
OMT decreases the AKT/mTOR signaling pathway and increases autophagy in chondrocytes treated with IL‑1β. (**a**–**e**) Western blot analysis and quantitative analysis of p-AKT, AKT, p-mTOR, mTOR, p62, and LC3 in chondrocytes. (**f**) LC3 expression is detected using the immunofluorescence assay. Data are presented as the mean ± SD, *n* = 3/group. **P < 0.01, *P < 0.05 versus Con group; ^##^*P* < 0.01, ^#^*P* < 0.05 versus IL-1β group

The AKT/mTOR signaling pathway is intricately linked to apoptosis and autophagy in chondrocytes. Western blot analysis was performed to assess the levels of AKT and mTOR in chondrocytes in each experimental group. The results showed that IL-1β increased the ratio of p-AKT/AKT and p-mTOR/mTOR, and OMT inhibited this increase. In addition, IL-1β decreased the ratio of LC3 II:I and increased the level of p62, while OMT exerted the opposite effect (Fig. 5A-E). This pattern was also observed for the fluorescence intensity of LC3(Fig. 5F). Collectively, these findings suggested that OMT played a protective role against osteoarthritis by activating autophagy.

### Effectiveness of OMT in improving chondrocyte viability under IL-1β treatment is limited in the presence of the autophagy inhibitor 3-MA

**Fig. 6:**
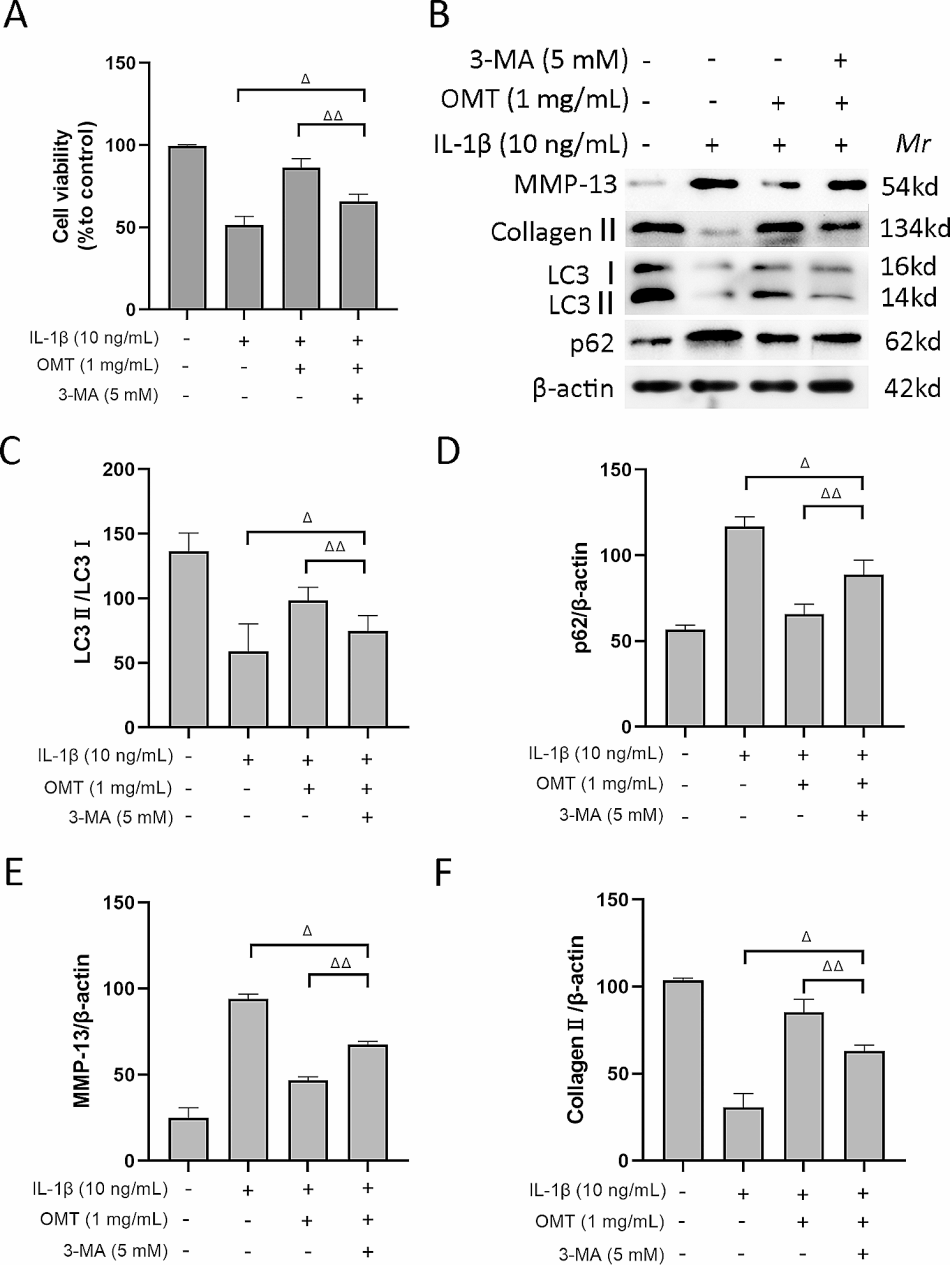
Role of autophagy in OMT protection. (**a**) The cell viability of chondrocytes detected by MTT assay. (**b-f**) Western blot analysis and quantitative analysis of MMP-13, collagen II, p62, and LC3. Data are presented as the mean ± SD. A: *n* = 9/group; B-F: *n* = 3/group. ^*△*^*P* < 0.05; ^*△△*^*P* < 0.01

To further verify the role of autophagy in the protective effects of OMT, we performed experiments using 3-MA, a specific autophagy inhibitor. The results of the cell viability assay revealed that chondrocyte viability was significantly lower in the IL-1β + OMT + 3-MA group than in the IL-1β + OMT group (*P* < 0.01). However, chondrocyte viability was significantly higher in the IL-1β + OMT + 3-MA group than in the IL-1β group (*P* < 0.05) (Fig. 6A, B). These findings indicated that 3-MA effectively inhibited the protective effect of OMT in these cells. Additionally, Western blot analysis of the ECM and autophagy-related proteins demonstrated that the IL-1β + OMT + 3-MA group had a lower LC3 II:I ratio and a higher p62 level than the IL-1β + OMT group, suggesting that 3-MA could decrease autophagy (Fig. 6C, D). Furthermore, compared to the IL-1β + OMT group, the IL-1β + OMT + 3-MA group showed higher MMP-13 protein expression and lower collagen II expression (Fig. 6E, F), indicating that 3-MA significantly diminished the beneficial effects of OMT on the ECM.

## Discussion

The mechanism by which OMT may regulate the pathogenesis of OA is unclear. The current study found that OMT effectively alleviated IL-1β-induced damage in chondrocytes. The underlying mechanism involved the activation of autophagy through inhibition of the AKT/mTOR pathway in chondrocytes. These findings provide baseline evidence for future applications of OMT in OA treatment.

IL-1β plays a significant role in the development of OA by promoting the degradation of the ECM of articular cartilage [31–33]. Furthermore, IL-1β is a key factor in triggering chondrocyte apoptosis, making it widely utilized as an apoptosis-inducing agent for chondrocyte studies [34, 35]. Chondrocytes treated with IL-1β offer a valuable in vitro model for studying OA chondrocytes [36, 37]. In OA, apoptosis and degradation of the chondrocyte ECM are significant pathological events [5]. Apoptosis has been observed in OA cartilage, indicating its role in the development of the disease [38]. Apoptosis is associated with cartilage damage and reduced cell density [39]. Therefore, apoptosis is a potential target for OA treatment, and understanding apoptosis is crucial for the development of new therapeutic strategies [40, 41].

Irreversible degradation of ECM is a central aspect of the pathological process of OA [42]. Chondrocytes are involved in ECM biosynthesis and collagen II degradation, which are important signals in OA [43, 44]. MMP-13 is the primary enzyme that contributes to cartilage degradation by cleaving type II collagen [45, 46]. The current study demonstrates that OMT significantly enhances the survival of chondrocytes, reduces apoptosis rates, and prevents IL-1β-induced degradation of the cartilage matrix. These findings suggest that OMT possesses a protective effect against chondrocyte damage induced by IL-1β.

Oxidative stress significantly contributes to the development of osteoarthritis. An imbalance between ROS production and the antioxidant capacity of chondrocytes leads to cartilage degradation and chondrocyte apoptosis [47–50]. In the current study, IL-1β was utilized to induce damage to chondrocytes, mimicking the cellular model of OA. The experimental results demonstrate that OMT has an inhibitory effect on IL-1β-induced elevation of ROS in chondrocytes, thereby suggesting that OMT may provide a protective effect by suppressing oxidative stress. Excessive ROS levels not only lead to oxidative damage but also disrupt cell signaling pathways, including the AKT/mTOR pathway [51]. The AKT/mTOR pathway, which involves more than 150 proteins, plays a crucial role in maintaining joint health and is thus involved in OA development [52, 53]. This study found that OMT effectively reduced the IL-1β-induced activation of the AKT/mTOR signaling pathway. This suggests that OMT exerts its effects by inhibiting the AKT/mTOR pathway.

mTOR is a crucial suppressor of autophagy and is primarily regulated by upstream signaling molecules involving AKT [53–55]. Autophagy, a vital regulator of energy utilization and nutrient metabolism, is involved in cellular homeostasis by eliminating dysfunctional and damaged macromolecules and organelles [56]. Autophagy failure can result in death at the cellular level [56]. The transition from autophagy to apoptosis plays a significant role in the progression of chondrocytes to OA [57]. mTOR upregulation in the OA cartilage is associated with increased chondrocyte apoptosis and reduced expression of autophagy-related genes. In mice, the cartilage knockdown of mTOR results in elevated autophagy, decreased apoptosis, and altered cartilage homeostasis [58]. Administration of the mTOR inhibitor rapamycin mitigates the severity of experimental OA by stimulating autophagy [59].

LC3 conversion (i.e., from LC3-I to LC3-II) reflects the progression of autophagy, and LC3 detection by immunoblotting is often used to monitor autophagic activity [60]. The p62 protein, also called sequestosome 1, is degraded during autophagy and serves as a marker for studying autophagic flux [61]. The present study found that IL-1β suppressed the level of autophagy as indicated by the reduced ratio of LC3 II:I and increased protein level of p62 in chondrocytes. After treatment with OMT, the levels of autophagy were enhanced in IL-1β-treated chondrocytes.

In further experiments using the autophagy inhibitor 3-MA to verify the role of autophagy in OMT protection, the effect of OMT on chondrocyte autophagy was significantly weakened after the addition of 3-MA. This was mainly manifested as a decreased ratio of LC3 II:I and an increased level of p62. Simultaneously, the protective effect of OMT on chondrocytes was significantly weakened, and this was mainly reflected as decreased rate of cell survival, increased protein level of ECM MMP-13, and decreased level of collagen II. These results suggest that autophagy plays an important role in OMT protection. We also notice that Autophagy serves dual roles in OA, acting both to protect cells and promote cell death [62, 63]. It can influence the survival and death of chondrocytes during different stages of osteoarthritis progression [62, 64]. So, we need to use difference model representing different stages of OA to demonstrate the effectiveness of OMT. This will help to identify candidates for benefiting from OMT treatment.

In summary, IL-1β can induce chondrocyte apoptosis and decrease ECM synthesis, increase ROS production, activate the AKT/mTOR pathway, and inhibit autophagy. OMT can alleviate chondrocyte apoptosis and ECM synthesis induced by IL-1β. The mechanism may be related to the inhibition of ROS production, inhibition of the AKT/mTOR pathway, and activation of autophagy, suggesting that OMT can be used to treat OA.

Our study had some limitations. First, the data obtained from in vitro experiments may differ from those obtained from in vivo experiments. Therefore, the curative effect of OMT in OA requires further investigation. Second, Studying the effects of combining 3-MA and OMT on the AKT-mTOR signaling pathway would improve the paper’s clarity.

## Conclusions

OMT has a protective effect against IL-1β-induced chondrocyte damage. The potential mechanism involves the reduction of oxidative stress and inhibition of the AKT/mTOR signaling pathway, thereby promoting autophagy. These findings suggest that OMT is a promising and effective therapeutic option for the clinical management of OA.

## Data Availability

The datasets used and/or analyzed during the current study are available from the corresponding author on reasonable request.

## Abbreviations

ECM: Extracellular matrix
IL-1β: Interleukin-1β
MMP-13: Matrix metalloproteinase-13
OA: Osteoarthritis
OMT: Oxymatrine
PBS: Phosphate-buffered saline
ROS: Reactive oxygen species
